# Immunological surveillance using anti-gSG6-P1 IgG biomarker reveals spatio-temporal dynamics of *Anopheles* exposure and gaps in malaria risk assessment in the Greater Mekong Subregion

**DOI:** 10.1051/parasite/2026012

**Published:** 2026-03-13

**Authors:** Manop Saeung, Natapong Jupatanakul, Niramon Jampeesri, Aneta Afelt, Theeraphap Chareonviriyaphap, Sylvie Manguin

**Affiliations:** 1 Department of Entomology, Faculty of Agriculture, Kasetsart University Ngamwongwan Road Bangkok 10900 Thailand; 2 HSM, University of Montpellier, CNRS, IRD 15 Avenue Charles Flahault Montpellier 34093 France; 3 Department of Medical Entomology, Faculty of Tropical Medicine, Mahidol University Ratchawithi Road Bangkok 10400 Thailand; 4 National Center for Genetic Engineering and Biotechnology (BIOTEC) Phahon Yothin Road Pathum Thani 12120 Thailand; 5 Vector-borne Disease Control Unit (10.1.3), Division of Vector Borne Diseases, Department of Disease Control, Ministry of Public Health Krung Thai Road Sisaket 33150 Thailand; 6 Interdisciplinary Centre for Mathematical and Computational Modelling, University of Warsaw 26/28 Krakowskie Przedmiescie Warsaw 00-927 Poland; 7 Espace-DEV, French National Research Institute for Sustainable Development 500 rue Jean-François Breton Montpellier 34393 France; 8 Research and Lifelong Learning Center for Urban and Environmental Entomology, Kasetsart University Institute for Advanced Studies, Kasetsart University Ngamwongwan Road Bangkok 10900 Thailand; 9 Royal Society of Thailand Chaeng Watthana Road Bangkok 10210 Thailand

**Keywords:** *Anopheles* mosquitoes, Immunological biomarker, Human activity, Rubber plantation, Sisaket Province, Thai-Cambodia border, Malaria risk assessment

## Abstract

Entomological parameters such as mosquito biting rates often fail to capture variability in human behavior, thereby limiting its accuracy for assessing the population-level malaria risk. This study investigated the use of previously described *Anopheles gambiae*-based anti-salivary biomarker, anti-gSG6-P1, as a serological marker for *Anopheles* exposure, and examined key entomological, human, and environmental risk factors in Sisaket Province, Thailand. Blood samples were collected via finger prick from the same set of 184 participants across three seasons: rainy (August 2022), cool-dry (December 2022), and hot-dry (April 2023). Anti-gSG6-P1 IgG levels were quantified using ELISA. Factor Analysis of Mixed Data revealed that seasonality exerted the strongest influence on anti-gSG6-P1 IgG levels, which was likely driven by human activities, particularly the frequency of rubber tapping activity in the areas where *Anopheles dirus* is present. A higher frequency of rubber plot entry (5–7 days/week) significantly increased anti-gSG6-P1 IgG responses (1.08 ± 0.36) compared with the lower frequency group (0–4 days/week) (0.96 ± 0.35). Furthermore, our findings revealed the complex interplay between anti-gSG6-P1 IgG levels and the seasonality of human behavioral and vector dynamics. These factors highlight key limitations of the anti-gSG6-P1 IgG biomarker in the Greater Mekong Subregion, particularly the lack of well-characterized anti-gSG6-P1 IgG serological response kinetics in regions where predominant vector species exhibit low salivary peptide homology to *An. gambiae*. These findings emphasize the need for new serological tools tailored to malaria vector species present in the Subregion to improve malaria risk assessment and strengthen vector control strategies.

## Abstract (fr)

Les paramètres entomologiques tels que le taux de piqûres de moustiques ne permettent souvent pas de saisir la variabilité du comportement humain, ce qui limite leur précision pour évaluer le risque de paludisme au niveau de la population. Cette étude examine l’utilisation du biomarqueur antisalivaire anti-gSG6-P1, précédemment décrit et basé sur *Anopheles gambiae*, comme marqueur sérologique de l’exposition aux anophèles, et examine les principaux facteurs de risque entomologiques, humains et environnementaux dans la province de Sisaket, en Thaïlande. Des échantillons sanguins ont été prélevés par piqûre à la pulpe du doigt sur le même groupe de 184 participants au cours de trois saisons : pluvieuse (août 2022), fraîche et sèche (décembre 2022) et chaude et sèche (avril 2023). Les taux d’IgG anti-gSG6-P1 ont été quantifiés par des tests ELISA. L’analyse factorielle des données mixtes a révélé que la saisonnalité exerce la plus forte influence sur les taux d’IgG anti-gSG6-P1, probablement en raison des activités humaines, en particulier la fréquence de la récolte du caoutchouc dans les zones où *Anopheles dirus* est présent. Une fréquence plus élevée d’entrée dans les plantations d’hévéas (5 à 7 jours par semaine) augmente considérablement les réponses anti-gSG6-P1 IgG (1,08 ± 0,36) par rapport au groupe à fréquence plus faible (0 à 4 jours par semaine) (0,96 ± 0,35). De plus, nos résultats révèlent l’interaction complexe entre les taux d’IgG anti-gSG6-P1 et la saisonnalité du comportement humain et de la dynamique des vecteurs. Ces facteurs mettent en évidence les principales limites du biomarqueur anti-gSG6-P1 IgG dans la sous-région du Grand Mékong, en particulier l’absence de cinétique de réponse sérologique anti-gSG6-P1 IgG bien caractérisée dans les régions où les espèces vectrices prédominantes présentent une faible homologie peptidique salivaire avec *An. gambiae*. Ces résultats soulignent la nécessité de disposer de nouveaux outils sérologiques adaptés aux espèces vectrices d’agents du paludisme présentes dans la sous-région afin d’améliorer l’évaluation des risques de paludisme et de renforcer les stratégies de lutte antivectorielle.

## Introduction

Malaria remains a major global health problem, with the World Health Organization (WHO) estimating between 225 (2014) and 282 (2024) million annual cases and over 600,000 deaths per year between 2000 and 2024 [55]. The WHO’s malaria control initiatives during the past decades, targeting vectors and parasites [50, 51], have resulted in a significant decline in malaria incidence [53]. Consequently, low-transmission regions have transitioned towards elimination programs, tailoring interventions to local environments, as well as human and mosquito behavior with the involvement of communities [51].

Intensive control measures in the Greater Mekong Subregion (GMS), which includes Cambodia, China’s Yunnan Province, Laos, Myanmar, Thailand, and Vietnam, have significantly lowered regional malaria cases since 2015, leading to an elimination target set for 2030 [13, 49]. Although GMS cases dropped below 100,000 in 2020–2021 [52], recent years saw resurgence, surpassing 150,000 in 2022 and reaching approximately 225,000 in 2023, primarily driven by cases in Myanmar due to political instability [54].

Another critical challenge for malaria elimination in the GMS is outdoor transmission, which limits the effectiveness of standard indoor interventions such as insecticide-treated nets (ITNs) and indoor residual spraying (IRS) [14, 48]. Outdoor transmission primarily affects high-risk populations, including forest goers and rubber tappers, complicating the dynamics of residual transmission and making it difficult to identify focal areas or populations for targeted interventions [3, 14].

The entomological inoculation rate (EIR) is used to quantify malaria transmission intensity defined as the product of the human-biting rate and the proportion of *Anopheles* mosquitoes harboring *Plasmodium* sporozoite-stage parasites. However, in the GMS region, human–vector contact is heterogeneous across populations, especially influenced by microgeographic patterns of exposure, human behavioral heterogeneity, and mobility [9, 10, 14, 36, 38, 39]. Furthermore, primary transmission foci in the GMS, particularly forested and peri-forest habitats, are logistically difficult to access and often under-sampled, introducing substantial challenges to site representativeness and increasing the likelihood of sampling bias. These limitations contribute to considerable uncertainty in biting-rate-based estimates of transmission risks [37]. Moreover, human vulnerability varies based on occupation, location, and socioeconomic status [36, 41, 59], highlighting the need for alternative or complementary risk-assessment methods capable of capturing the complexity of transmission landscapes in the GMS.

To overcome the sampling constraints associated with biting-rate, one promising approach is the use of serological responses to *Anopheles* salivary gland proteins injected during mosquito bites, which serve as biomarkers of human exposure to bites. Among these, the most well-studied is the gSG6 protein family, named after the *Anopheles gambiae* salivary gland protein 6 [20], identified through transcriptome analyses and exclusively expressed in the salivary glands of adult female *An. gambiae* [12, 18, 20]. In Africa, gSG6 shows strong sequence conservation and broad binding across both vector and non-vector *Anopheles* species, a shorter derivative – the gSG6-P1 peptide – was developed and first evaluated as a biomarker for malaria vector species, *An. gambiae*, in sub-Saharan Africa [28]. A positive correlation was demonstrated between anti-gSG6-P1 IgG levels and exposures to bites of *An. gambiae*, suggesting its utility as a proxy for malaria vector bites [25, 28, 32, 33]. Beyond Africa, this peptide has also been evaluated in the Americas, where the key malaria vectors are not *An. gambiae* but rather *An. darlingi*, *An. albimanus*, and *An. nuneztovari*, and it similarly showed positive associations with mosquito biting pressure [21]. Furthermore, this peptide was validated in Asia, specifically in Tak Province, Thailand – an area with high malaria prevalence where *Anopheles minimus* is the dominant vector species, suggesting its broader applicability in the GMS [57]. However, malaria vectors in Thailand and the GMS are highly diverse, with at least seven important vector species commonly recognized, including *An. dirus*, *An. baimaii*, *An. minimus*, *An. maculatus*, *An. sawadwongporni*, *An. pseudowillmori*, and *An. aconitus* [44]. Therefore, further validation is required in other regions where different *Anopheles* vector species predominate.

This study aimed to evaluate the gSG6-P1 peptide as a serological marker for *Anopheles* exposure in a malaria transmission area along the Thailand-Cambodia border. Transmission in this region is characterized by outdoor and forest-associated biting, with *An. dirus* serving as the predominant vector species [37]. To evaluate gSG6-P1 performance under these ecological conditions, we quantified anti-gSG6-P1 IgG responses among populations at elevated risk of vector contact, particularly rubber tappers in Sisaket Province over the rainy (August 2022), cool-dry (December 2022), and hot-dry (April 2023) seasons. We further examined associations between anti-gSG6-P1–specific IgG levels and human behavioral, entomological, and environmental factors to determine the peptide’s effectiveness in reflecting *Anopheles* exposure and identifying key determinants of biting risk in the Thailand-Cambodia border area. This research seeks to improve our understanding of malaria transmission dynamics and establish a reliable serological tool for monitoring transmission risks in the GMS.

## Materials and methods

### Ethics

The study received ethics approval from the Ethics Review Committee for Research Involving Human Participants at Kasetsart University (Certificate of Approval No. CAO63/035). Formal written ethics clearance for the study protocol and written informed consent from volunteers were obtained before the trials commenced.

### Study locations

**Figure 1:**
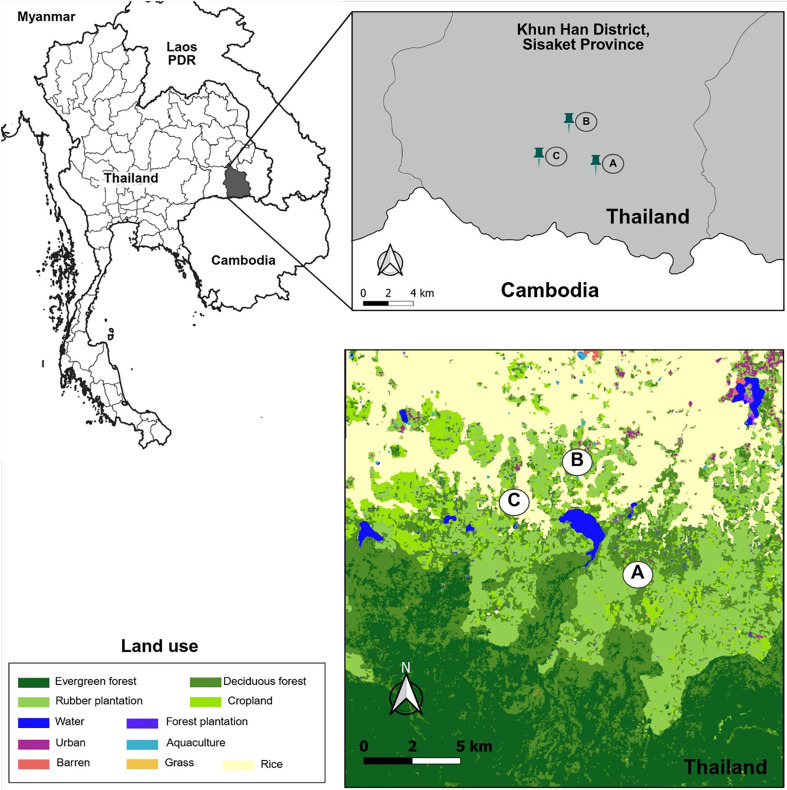
Study locations in Khun Han District, Sisaket Province, Northeastern Thailand. Three villages were selected, including Huai Chan (A), Kan Throm Tai (B), and Non Thong Lang (C).

Three villages (Huai Chan, Kan Throm Tai, and Non Thong Lang) located in the Khun Han District, Sisaket Province, were chosen due to the histologically high malaria burden (Fig. 1). These villages are gradually slope up towards the southern part of the province, where large rubber plantations and primary forests are located. Kan Throm Tai and Non Thong Lang are situated in lower areas (~200 m), surrounded by paddy fields and scattered rubber plantations. These two villages differ in their distance from the forest fringe, with Non Thong Lang being approximately 4 km closer to the forest fringe than Kan Throm Tai. In contrast, Huai Chan Village is located at a higher altitude (~240 m) and is mostly surrounded by rubber plantations, croplands, and deciduous forests. The southernmost area of Sisaket Province shares a border with Cambodia (Fig. 1). Agriculture is the main occupation of villagers in these areas. Weather conditions are categorized into three seasons, which are rainy (May–October), cool-dry (October–February), and hot-dry (February–May) [56]. Spatial data were obtained from the USAID website (https://landcovermapping.org/en/landcover/) and processed using QGIS software (version 3.22.7).

### Study design and inclusion criteria

A one-year cross-sectional survey was designed to capture the temporal dynamics of the anti-gSG6-P1 peptide from local people in at-risk groups. Finger prick blood samples were collected once per season, which started with the rainy (August 2022), cool-dry (December 2022), and hot-dry (April 2023) seasons. Participants were selected based on the following criteria: 1) age between 18–60 years old, 2) residing in these villages, 3) Thai citizenship, and 4) work in rubber plots. Each participant was followed up throughout the three seasons. A participant was considered a dropout if they were unable to provide a blood sample during either season.

### Dry blood spot collection and interviews

Finger prick blood was collected by local health authorities using Whatman No. 1 filter papers (Whatman International Ltd., Banbury, UK), size 3 × 12 cm, following an approved protocol. Two blood spots were collected from each participant and labeled with a unique identification code. Each blood sample was dried before being kept in a plastic zip-lock bag to prevent contamination. All blood samples were then shipped to the National Center for Genetic Engineering and Biotechnology (BIOTEC), Bangkok and stored at −20 °C to minimize protein degradation [4, 7] until further analysis.

To characterize the study population and identify factors potentially influencing anti-salivary IgG responses, information on participants’ engagement in rubber plantations was collected. Because season affects occupational and behavioral patterns, participants were asked during each blood collection to report their average weekly frequency of rubber plantation visits. Additionally, because participants’ residence and rubber plantation were not always co-located, we hypothesized that individuals living closer to plantation areas – and therefore nearer to forest fringes – might experience higher *Anopheles* exposure. To assess this potential spatial determinant of risk, we inquired on the distance between each participant’s residence and their associated rubber plantation.

### Anti-gSG6-P1 IgG detection by enzyme-linked immunosorbent assay (ELISA)

The immunological assay to detect human IgG against gSG6-P1 *Anopheles* salivary peptide used in this study was adapted from a previous protocol [57]. In brief, dry blood spot samples were cut into a circle shape (diameter: 6 mm) and individually eluted in 250 μL of 0.1% Tween (Cat# BP337-100, Thermo Fisher Scientific, Waltham, MA, USA) in 1× phosphate-buffered saline (1× PBS) at 4 °C for 48 h. Then, the filter paper was removed and eluted blood was divided into three tubes (~80 μL/tube) before use to reduce degradation from the freeze-thaw process. All samples were kept at −20 °C for further analysis with ELISA. The details of ELISA assay in this study are described in the Supplementary Methods.

### Statistical analysis and visualizations

The adjusted optical density (OD) values from all ELISA samples were analyzed to identify quantitative and qualitative factors influencing variability of anti-gSG6-P1 IgG responses using Factor Analysis for Mixed Data (FAMD) with the FactoMineR package [19] in RStudio (version 4.2.1). The details of FAMD analysis are mentioned in the Supplementary Methods.

Statistical comparisons of the IgG levels between two independent groups were performed using the Wilcoxon Rank-Sum Test, while comparisons between two dependent groups were conducted using the Wilcoxon Signed-Rank Test. Statistical significance was defined as a *p*-value <0.05. The data were analyzed using jamovi 2.3.6 (jamovi.org) and data visualization was performed in the RStudio program (version 4.2.1).

## Results

### Population characteristics

A total of 184 participants, aged 18–60 years from three villages, who provided blood samples across all three seasons, were included in data analyses. Most participants were from Kan Throm Tai (*n* = 97; 52.72%), followed by Huai Chan (*n* = 59; 32.07%), and Non Thong Lang (*n* = 28; 15.22%). Females constituted a larger proportion (*n* = 116; 63.05%) than males (*n* = 68; 36.95%), and this pattern was consistent across all three villages (Supplementary Tables 1 and 2). This may be due to differences in participant availability or willingness to participate, with females generally being more represented than males. Participants were categorized into ten-year age intervals within the population aged 18–60 years old, with the majority (*n* = 124; 67%) falling into two active age groups: 41–50 (*n* = 63; 34%) and 51–60 years old (*n* = 61; 33%). The distance between rubber plots and participants’ homes was classified into three groups: <1 km, 1–5 km, and >5 km. Most participants lived more than 5 km away (*n* = 102; 55.43%), followed by 1–5 km (*n* = 41; 22.30%), and less than 1 km (*n* = 6; 3.62%). Rubber plot entry activity varied by season, with higher frequency (5–7 days/week) reported in the cool-dry (*n* = 104; 56.5%) and hot-dry (*n* = 99; 53.8%) seasons compared to the rainy season (*n* = 55; 29.9%) (Supplementary Table 1).

### Exploring key variables influencing anti-gSG6-P1 IgG responses using FAMD

**Figure 2:**
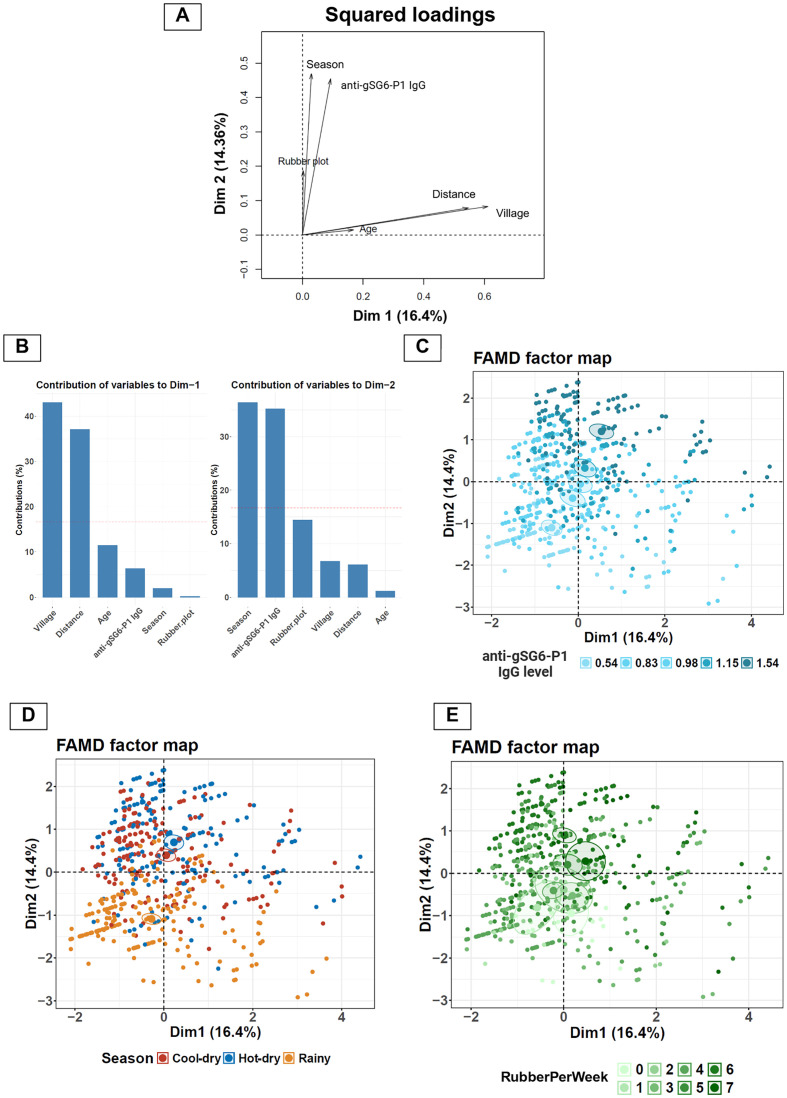
FAMD analysis reveals underlying variables influencing the level of anti-gSG6-P1 responses. (A) Squared loadings plot demonstrating the correlations between categorical and continuous variables with Dims 1 and 2. (B) Plots demonstrating contributions of each variable to Dims-1 and -2. (C–E) FAMD factor maps represents coordinates of each sample on the Dims-1 and -2, with color overlays indicating (C) IgG response levels, (D) season, and (E) frequency of rubber plot entry per week (RubberPerWeek).

To identify the key factors influencing anti-gSG6-P1 IgG levels, we conducted FAMD to explore relationships between population characteristics and antibody levels. This analysis explored the relationships between population characteristics (season, frequency of entering rubber plots per week, age, village, and distance from residence to rubber plot) and individual anti-gSG6-P1 IgG levels. The initial step in FAMD involves the computation of principal dimensions (Dims), which consolidate the variance from the original variables, thereby simplifying the interpretation of complex inter-variable relationships within the dataset. The proportion of total variance in the dataset was explained by each Dim. Dims 1 and 2 collectively accounted for only 30.76% of the total variance, indicating a high level of complexity in the dataset and this suggests that multiple factors contribute to the observed variations in IgG levels. The contribution of each variable to the principal dimensions was demonstrated by squared loading plots (Figs. 2A and 2B). Anti-gSG6-P1 IgG levels demonstrated a stronger association with Dim 2, which was primarily influenced by season (36% contribution) and the frequency of entering rubber plots per week (RubberPerWeek; 35% contribution). This suggests these two factors are major determinants of anti-gSG6-P1 levels. Dim 1 exhibited a lesser influence on anti-gSG6-P1 IgG levels, with village (43% contribution) and distance from residence to the rubber plot (37% contribution) being its main contributors to the dimension.

Based on these findings, three key variables – IgG levels, season, and RubberPerWeek – were selected for further analysis to assess their directional relationships. The color overlay on individual data points of the FAMD map revealed the relationship among variables and the IgG levels. Individuals with low anti-gSG6-P1 IgG levels cluster in the lower left (−, −) quadrant and those with high anti-gSG6-P1 IgG levels cluster in the upper right (+, +) quadrant (Fig. 2C). The color overlay for season showed that the rainy season corresponded to lower anti-gSG6-P1 IgG levels, while the cool-dry and hot-dry seasons were associated with higher anti-gSG6-P1 IgG levels (Fig. 2D). Furthermore, color overlay for RubberPerWeek revealed that increased entries into rubber plots corresponded to the individuals with higher anti-gSG6-P1 IgG levels observed during the cool-dry and hot-dry seasons (Fig. 2E). Conversely, lower anti-gSG6-P1 IgG levels during the rainy season were associated with low RubberPerWeek frequencies. These findings underline a relationship between seasonal rubber plot activity and IgG responses (Fig. 2E).

### Effect of rubber plot visit frequency on anti-gSG6-P1 IgG levels

**Figure 3:**
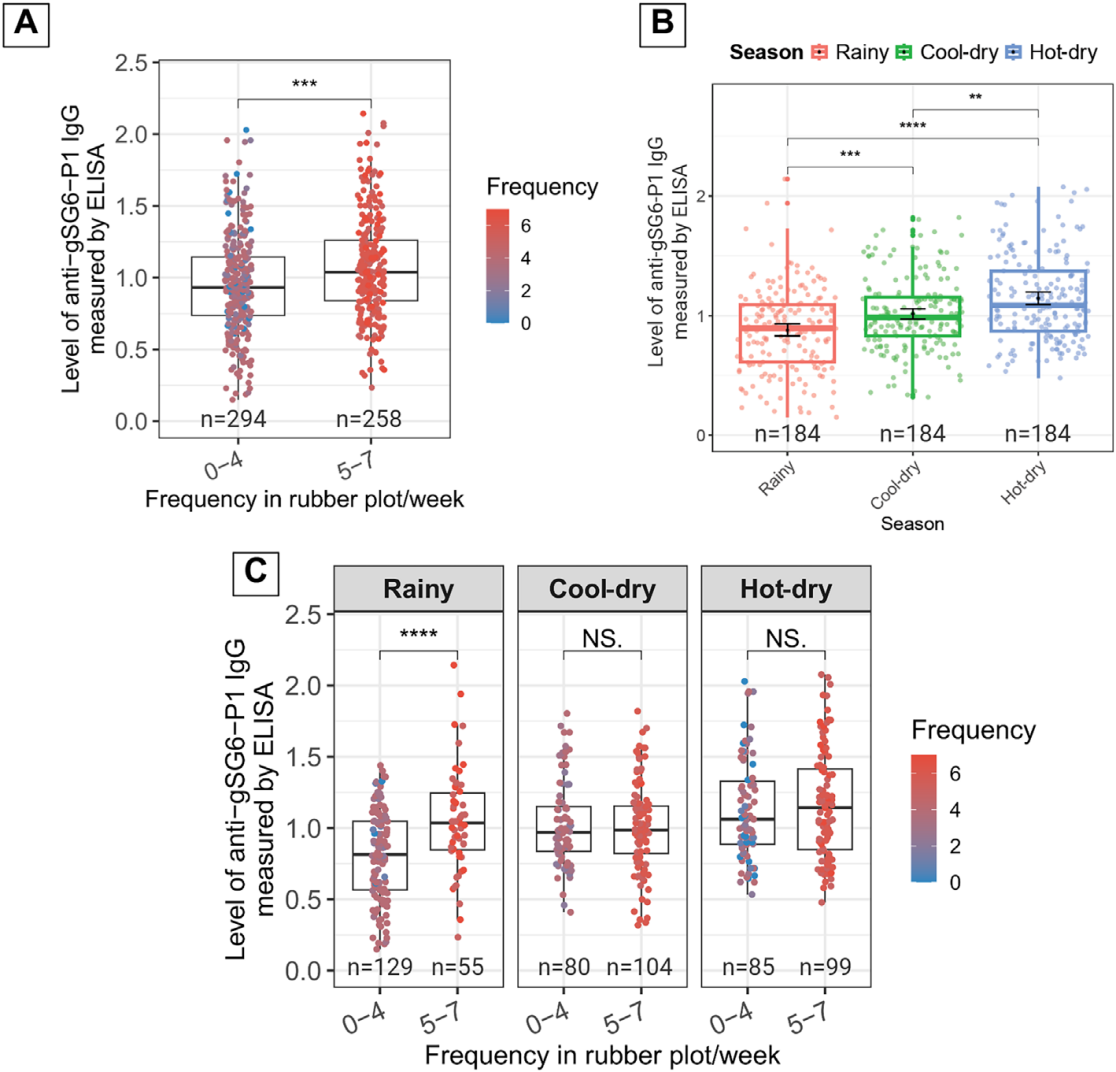
Frequency of entering the rubber plot/week and seasonal variables affecting anti-gSG6-P1 response. (A) Box plot comparing levels of anti-gSG6-P1 between participants with low- and high-rubber plot visit frequency. Higher IgG responses were associated with more frequent visits to the rubber plot, regardless of seasonal variation. (B) Box plot demonstrating temporal dynamics of IgG responses across three seasons. (C) Box plot demonstrating the combined effect of season and rubber plot entry frequency on anti-gSG6-P1 IgG levels. The box plot displays the interquartile range, and the whiskers indicate the range of maximum and minimum values. The dot plots display the raw data from each individual. Statistical analyses were performed using the Wilcoxon Rank-Sum Test, with significance levels indicated as *p* < 0.05 (*), *p* < 0.01 (**), *p* < 0.001 (***), *p* < 0.0001 (****), while NS indicates no statistically significant *p* > 0.05.

While FAMD identified frequency of rubber plots entry as one of the factors correlated with anti-gSG6-P1 IgG levels, it did not provide a direct statistical comparison between participants with varying visit frequencies. Since the participants with visit frequency between 0–4 times per week were clustered together in the bottom part of the FAMD map (Fig. 2E), we separated participants based on rubber plot visit frequency into two groups, high (5–7 days/week) and low (0–4 days/week) frequency, then compared the anti-gSG6-P1 IgG levels between these groups. We found a significant increase in anti-gSG6-P1 IgG levels among the participants with high (1.08 ± 0.36) compared to low (0.96 ± 0.35) frequency of rubber plot entry (Fig. 3A).

To further explore the temporal dynamics of the anti-gSG6-P1 IgG responses, we also analyzed seasonal variations in anti-gSG6-P1 IgG levels, which were the lowest during the rainy season (0.88 ± 0.34), increased during the cool-dry season (1.02 ± 0.31), and peaked during the hot-dry season (1.15 ± 0.36) (Fig. 3B). When considering both frequency and seasonal variations, distinct anti-gSG6-P1 IgG level patterns between the high- and low-frequency groups over different seasons were observed. During the rainy season, individuals with a high frequency of rubber plot entry had significantly elevated anti-gSG6-P1 IgG levels (1.05 ± 0.37) compared to those with low entry frequency (0.81 ± 0.31). In contrast, during the cool-dry season, the anti-gSG6-P1 IgG levels in the low-frequency group increased to 1.03 ± 0.30, reaching a level comparable to the high-frequency group, which remained unchanged at 1.01 ± 0.31. During the hot-dry season, the IgG levels were elevated across both groups, with high-frequency individuals reaching 1.16 ± 0.38 and low-frequency individuals at 1.12 ± 0.35 (Fig. 3C).

### Spatio-temporal dynamics of anti-gSG6-P1 IgG responses in the study population

**Figure 4:**
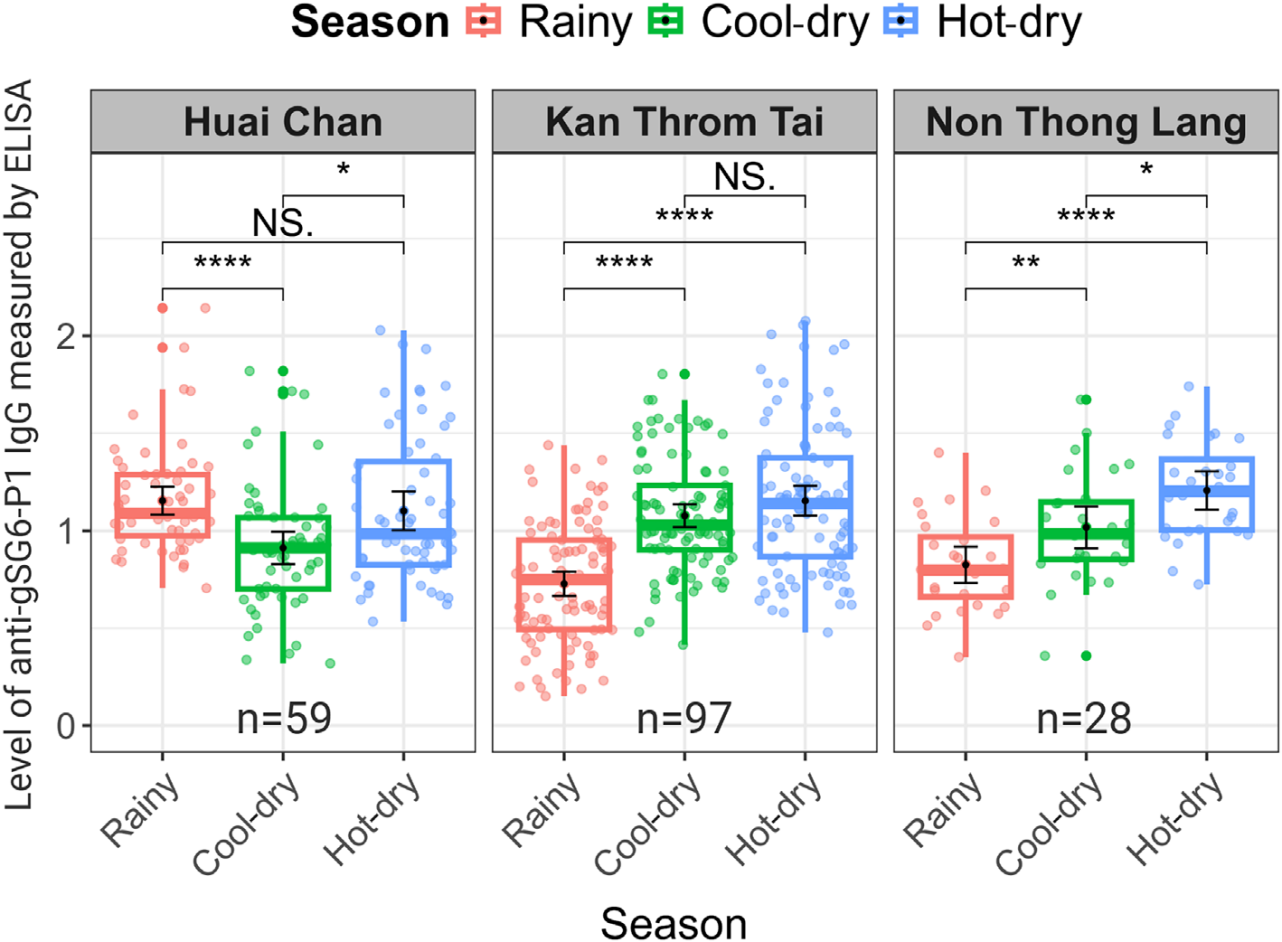
Spatial-temporal dynamics of IgG responses among all participants across three seasons in each village. The box plot displays the interquartile range, and the whiskers indicate the range of maximum and minimum values. The dot plots display the raw data from each individual. Statistical analyses were performed using the Wilcoxon Signed-Rank Test, with significance levels indicated as *p* < 0.05 (*) and *p* < 0.001 (***), *p* < 0.0001 (****), while NS indicates no significant difference *p* > 0.05.

When stratified by village, our analysis revealed similar patterns in two villages, Kan Throm Tai and Non Thong Lang. In both villages, the anti-gSG6-P1 IgG levels were lowest during the rainy season (0.73 ± 0.31 and 0.83 ± 0.24, respectively) and increased during the cool-dry season (1.08 ± 0.29 and 1.02 ± 0.28) and hot-dry season (1.15 ± 0.38 and 1.21 ± 0.25, respectively) (Fig. 4). In contrast, Huai Chan village had a distinct population IgG response pattern. The IgG levels were highest during the rainy season (1.15 ± 0.27), decreased to their lowest during the cool-dry season (0.91 ± 0.32), and then increased again during the hot-dry season (1.10 ± 0.38) (Fig. 4). This unique pattern of elevated IgG levels observed in Huai Chan during the rainy season may be due to a higher proportion of residents in Huai Chan frequently entering rubber plots during the rainy season (5–7 times per week) compared to the other two villages (Supplementary Table 1).

## Discussion

A longitudinal serological and behavioral survey conducted among rubber tappers in malaria-prone areas of Sisaket Province identified key factors influencing anti-gSG6-P1 IgG responses, including frequency of visits to rubber plantations and season. Entry into rubber plantation or forest ecotypes has previously been associated with increased malaria transmission risk, due to the prevalence of vectors in these ecotypes [34]. Consistent with this, our two-year longitudinal entomological survey (July 2022 to March 2024) in Khun Han District, Sisaket Province [37] revealed that the majority (72%) of *Anopheles* mosquitoes were collected from rubber-forest ecotypes, with *An. dirus* comprising 84% of captured mosquitoes [37]. Although *An. dirus* was present year-round, its abundance fluctuated seasonally starting from 24% in the rainy season, peaked at 68% during the cool-dry season (68%), and reduced to the lowest during the hot-dry (7%) seasons.

We found that anti-gSG6-P1 IgG responses do not solely reflect contemporaneous vector density. Rather, exposure risk appears to be shaped by a varying combination of entomological and human behavioral factors depending on the season. Anti-gSG6-P1 IgG responses were lowest during the rainy season (0.88 ± 0.34 normalized OD_410 nm_), coinciding with low rubber plot visitation rates (0–4 days/week for 129 participants, *versus* 5–7 days/week for 55 participants). Then, responses increased from the rainy season to the cool-dry season (1.02 ± 0.31), coinciding with both: peak *An. dirus* abundance and increased rubber tapping activity during the cool-dry season (5–7 days/week for 104 participants). However, responses remained elevated at 1.15 ± 0.36 during the hot-dry season despite low *Anopheles* abundance during this period, likely sustained by continued high-frequency rubber plot visits (5–7 days/week for 99 participants). Increased human activity in rubber plantations and forest-adjacent areas during periods of lower mosquito abundance in the hot-dry season may partially compensate for reduced vector density. Additionally, sustained high anti-gSG6-P1 IgG levels during the hot-dry season may reflect carry-over antibody responses induced by high exposure during the peak vector density from the preceding cool-dry season [35, 37]. This highlights the complex interplay between vector ecology and human behavior in shaping exposure risk, and more importantly, reveals important gaps in our understanding of the kinetics and persistence of anti-gSG6-P1 IgG responses, particularly following exposure to different *Anopheles* species.

Further analyses comparing anti-gSG6-P1 IgG levels between high- and low-frequency visit groups, combined with daily *Anopheles* biting rates [37], demonstrated that both entomological and behavioral factors drive temporal variations in *Anopheles* biting risk. During the rainy season, when *Anopheles* biting rates averaged 7 bites/person/night, participants entering rubber plots ≥5 days per week exhibited significantly higher anti-gSG6-P1 IgG levels than those with fewer visits. However, in the cool-dry season, when *Anopheles* biting rates peaked at 19.5 bites/person/night, the IgG levels did not significantly differ based on visit frequency. This suggests that even infrequent exposure may be sufficient to elicit an IgG response when mosquito density is high. However, this interpretation requires further validation as there is currently no available kinetic data on the number of mosquito bites required to elicit an IgG response.

**Supplementary Figure 1.**
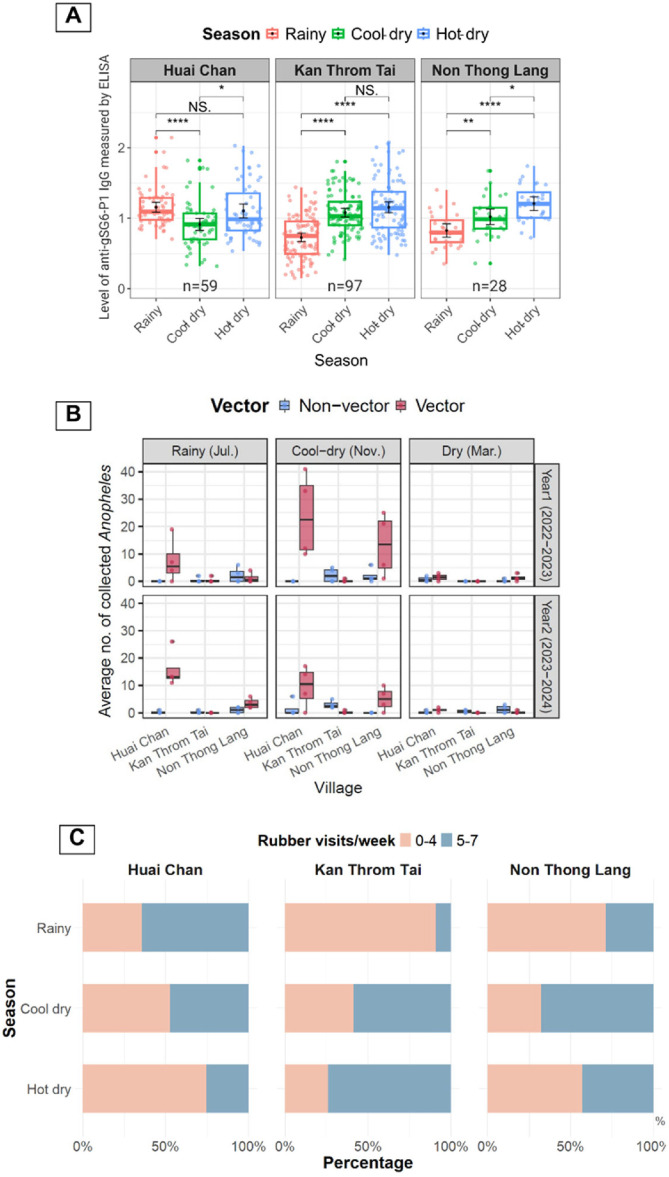
Spatio-temporal dynamics of anti-gSG6-P1 IgG, *Anopheles* mosquitoes, and human activity in rubber plot/week among three villages in Khun Han District, Sisaket Province; (A) An-anti-gSG6-P1 IgG responses among participants (reproduced from the main text to facilitate comparison with interacting factors), (B) Abundance of *Anopheles* mosquitoes collected from rubber-forest ecotype (Retrieved from Saeung et al. 2025: DOI: https://doi.org/10.1016/j.actatropica.2025.107545), and (C) Rubber plot visits per week among participants.

Although we observed a clear overall relationship between anti-gSG6-P1 IgG responses and the combined effects of entomological and human behavioral factors, village-level analyses revealed heterogeneity that could not be fully explained by the data collected in this study. In Kan Throm Tai and Non Thong Lang, anti-gSG6-P1 IgG levels were low during the rainy season and increased during the cool-dry and hot-dry seasons (Supplementary Figure 1A), consistent with periods of higher vector abundance (Supplementary Figure 1B) and/or increased rubber plot visitation (Supplementary Figure 1C), and in line with the overall trend. In contrast, Huai Chan exhibited a distinct pattern with elevated anti-gSG6-P1 IgG levels during the rainy season that may reflect an earlier seasonal increase in *An. dirus* density and high rubber forest visits in this village (Supplementary Figures 1B, 1C). Anti-gSG6-P1 IgG levels then decreased during the cool-dry season, aligning with lower rubber plot visitation rates (Supplementary Figure 1C). The elevated anti-gSG6-P1 IgG levels were observed in the hot-dry season despite lower vector abundance and rubber plot visitation rates (Supplementary Figures 1B, 1C), suggesting the influence of additional, unmeasured factors affecting exposure or serological responses. To better resolve these complex and site-specific dynamics, more detailed longitudinal studies with frequent entomological, behavioral, and serological data collections are required. Such research would enable a comprehensive assessment of the temporal dynamics of IgG responses in relation to human activity and mosquito abundance. Ultimately, these insights will improve our understanding of malaria transmission, particularly in the context of outdoor exposure, and will help refine vector control strategies.

Our study demonstrated limitation of the gSG6-P1 peptide specificity and sensitivity as a general biomarker for *Anopheles* bites. Although initially developed for *An. gambiae* exposure, the gSG6-P1 peptide appears to perform accurately in sub-Sahara African regions where *An. gambiae* is prevalent [2, 6, 11, 17, 27, 28, 33, 40]. However, inconsistent results have been reported from regions with limited or no *An. gambiae* presence [25, 57]. These variations suggest that while gSG6-P1 may serve as a biomarker for *Anopheles* exposure, its effectiveness varies significantly depending on the dominant local vector species. Our findings align with previous studies showing limited peptide sensitivity for certain species, including *Anopheles arabiensis* in Tanzania [17] and *Anopheles farauti* in the Solomon Islands [29]. Differences in specificity and sensitivity of salivary peptide biomarkers across regions are likely due to varying sequence similarities between local *Anopheles* species and the *An. gambiae* salivary peptide. For instance, the strong correlation observed in Tak Province, Thailand, was likely due to the dominance of *An. minimus*, whose salivary peptide shares 87% sequence similarity with the *An. gambiae* gSG6-P1 peptide [57]. Conversely, the weaker changes in gSG6-P1 IgG responses observed in our study area, where *An. dirus* dominates and shares only 47.8% salivary peptide similarity [57], underscores the peptide’s limited sensitivity in highly diverse vector regions such as the GMS [24, 31, 43]. Therefore, developing alternative serological biomarkers tailored specifically to prevalent local *Anopheles* species, especially main vectors such as *An. dirus*, is essential to accurately assess malaria transmission risks. Such biomarkers could be identified through comparative genomic, salivary glands transcript/proteomic analyses across *Anopheles* species, followed by immunogenicity screening of candidate proteins or peptides.

Given that sibling species within *Anopheles* complexes can vary widely in vector competence, from primary vectors to non-vectors [22], another key direction for future research is the development of species-specific anti-salivary biomarkers. The ability to differentiate exposure to bites from specific *Anopheles* species is critical to accurately assess transmission risk. Although this remains technically challenging – due to limited salivary peptide sequence data and high sequence similarity among peptides – achieving species-specific resolution is beneficial for guiding targeted and effective malaria control strategies.

With growing concerns over zoonotic simian malaria transmission in Thailand [5, 16, 23, 26], Malaysia [8, 30, 42, 45, 47], and other Southeast Asian countries [15], understanding the dynamics of transmission is crucial – particularly in identifying key transmission hotspots, as well as vectors and reservoirs involved. In these regions, several members of the Leucosphyrus group, including the *Anopheles leucosphyrus* and *An. dirus* complexes, act as bridge vectors, facilitating the transmission of simian malaria parasites to humans [1, 46, 58]. With slight modifications of the ELISA protocol, *Anopheles* species specific salivary biomarkers could serve as a valuable tool for assessing vector exposure in both humans and non-human primates such as macaques. When integrated with parasite surveillance in vectors and reservoirs, these biomarkers could provide critical insights into parasite-vector-host interactions, enhance malaria surveillance efforts, and support the development of more targeted control strategies for both human and zoonotic malaria transmission.

Our study highlights critical research gaps in the kinetics of anti-salivary IgG responses to *Anopheles* bites, which should be addressed for the reliable implementation of this biomarker as a surveillance tool. Key questions remain, including: i) the magnitude of anti-gSG6-P1 IgG response following mosquito bites at varying biting intensities, ii) how quickly anti-gSG6-P1 IgG levels increase after exposure to mosquito bites, iii) the decay rate of anti-salivary IgG antibodies over time, iv) how repeated exposure to mosquito bites affects the IgG response, and v) how anti-gSG6-P1 IgG responses vary after bites from different *Anopheles* species. Addressing these questions through controlled laboratory studies and subsequent field validations will provide better understanding of the dynamics of immune responses to *Anopheles* bites and improve the utility of salivary peptide-based biomarkers for malaria risk assessment and improve vector surveillance strategies.

## Conclusions and perspectives

This serological survey using an anti-gSG6-P1 salivary biomarker demonstrated that malaria transmission results from a complex interplay of human, vector, parasite, and ecological components, all of which can be influenced by seasonality. Focusing solely on one component of malaria transmission might be insufficient or inaccurate for interpreting risk. Our longitudinal study, which integrated immunological surveillance with entomological and human behavioral data, identified outdoor transmission zones associated with rubber-forest ecotypes as key areas of human–vector contact. While the gSG6-P1 salivary peptide has shown promise in identifying high-risk groups for malaria infection in the setting where *An. dirus* is present, the development and validation of species-specific salivary peptides biomarkers for other dominant *Anopheles* vectors is essential to improve the resolution and interpretability of immunological based surveillance. This is particularly important in regions where vector species composition varies, or where zoonotic simian malaria transmission is emerging. Importantly, the methodological framework and insights from this study are applicable beyond the Thai-Cambodia border. In other malaria-endemic areas with complex ecologies and heterogeneous vector populations, this integrative surveillance approach can support the identification of transmission hotspots, guide targeted interventions, and enhance the precision of malaria risk assessments across diverse geographic and epidemiologic settings.

## Data Availability

All data supporting the conclusions of this article are included within the article and supporting materials.

## Supplementary files

*Supplementary Methods*: Detailed protocols for gSG6-P1 Antibody detection by enzyme-linked immunosorbent assay (ELISA) and FAMD. *Supplementary Table 1*. Characteristics of the 184 participants from Sisaket Province (age 18–60 years old), from August 2022 to April 2023. *Supplementary Table 2*. Raw data containing characteristics and gSG6-P1 levels of each participant.

The supplementary material of this article is available at https://www.parasite-journal.org/10.1051/parasite/2026012/olm.

## References

[1] Boyer S, Doeurk B, Rakotonirina A, Chy S, Vong C, Piv E, Tat B, Ea M, Chhin C, Phen S, Kloeung N, Ke S, Popovici J, Piola P, Witkowski B, Maquart PO, Vantaux A. 2025. *Anopheles* mosquitoes in Mondulkiri forest, Cambodia: abundance, distribution, seasonal patterns and *Plasmodium* prevalence. Malaria Journal, 24(1), 6. 39794774 10.1186/s12936-024-05166-9 PMC11720960

[2] Brosseau L, Drame PM, Besnard P, Toto JC, Foumane V, Le Mire J, Mouchet F, Remoue F, Allan R, Fortes F, Carnevale P, Manguin S. 2012. Human antibody response to *Anopheles* saliva for comparing the efficacy of three malaria vector control methods in Balombo, Angola. PLoS One, 7(9), e44189. 23028499 10.1371/journal.pone.0044189 PMC3454387

[3] Carnevale P, Manguin S. 2021. Review of issues on residual malaria transmission. Journal of Infectious Diseases, 223(12 Suppl 2), S61–S80. 33906221 10.1093/infdis/jiab084 PMC8079138

[4] Chamchoy K, Praoparotai A, Pakparnich P, Sudsumrit S, Swangsri T, Chamnanchanunt S, Songdej D, Imwong M, Boonyuen U. 2021. The integrity and stability of specimens under different storage conditions for glucose-6-phosphate dehydrogenase deficiency screening using WST-8. Acta Tropica, 217, 105864. 33607062 10.1016/j.actatropica.2021.105864

[5] Chantaramongkol J, Buathong R. 2016. A fatal malaria caused by *Plasmodium knowlesi* infection in a healthy man, Betong, Yala, Thailand April, 2016. International Journal of Infectious Diseases, 53, 124.

[6] Cheteug G, Elanga-Ndille E, Donkeu C, Ekoko W, Oloume M, Essangui E, Nwane P, NS SE, Etang J, Wanji S, Ayong L, Eboumbou Moukoko CE. 2020. Preliminary validation of the use of IgG antibody response to *Anopheles* gSG6–p1 salivary peptide to assess human exposure to malaria vector bites in two endemic areas of Cameroon in Central Africa. PLoS One, 15(12), e0242510. 33382730 10.1371/journal.pone.0242510 PMC7774847

[7] Colson KE, Potter A, Conde-Glez C, Hernandez B, Ríos-Zertuche D, Zúñiga-Brenes P, Iriarte E, Mokdad AH. 2015. Use of a commercial ELISA for the detection of measles-specific immunoglobulin G (IgG) in dried blood spots collected from children living in low-resource settings. Journal of Medical Virology, 87(9), 1491–1499. 25988945 10.1002/jmv.24136

[8] Cooper DJ, Rajahram GS, William T, Jelip J, Mohammad R, Benedict J, Alaza DA, Malacova E, Yeo TW, Grigg MJ, Anstey NM, Barber BE. 2020. *Plasmodium knowlesi* malaria in Sabah, Malaysia, 2015–2017: ongoing increase in incidence despite near-elimination of the human-only *Plasmodium* species. Clinical Infectious Diseases, 70(3), 361–367. 30889244 10.1093/cid/ciz237 PMC7768742

[9] Cui L, Yan G, Sattabongkot J, Cao Y, Chen B, Chen X, Fan Q, Fang Q, Jongwutiwes S, Parker D, Sirichaisinthop J, Kyaw MP, Su XZ, Yang H, Yang Z, Wang B, Xu J, Zheng B, Zhong D, Zhou G. 2012. Malaria in the Greater Mekong Subregion: heterogeneity and complexity. Acta Tropica, 121(3), 227–239. 21382335 10.1016/j.actatropica.2011.02.016 PMC3132579

[10] Cui L, Yan G, Sattabongkot J, Chen B, Cao Y, Fan Q, Parker D, Sirichaisinthop J, Su XZ, Yang H, Yang Z, Wang B, Zhou G. 2012. Challenges and prospects for malaria elimination in the Greater Mekong Subregion. Acta Tropica, 121(3), 240–245. 21515238 10.1016/j.actatropica.2011.04.006 PMC3155744

[11] Drame PM, Machault V, Diallo A, Cornélie S, Poinsignon A, Lalou R, Sembène M, Dos Santos S, Rogier C, Pagès F, Le Hesran JY, Remoué F. 2012. IgG responses to the gSG6–P1 salivary peptide for evaluating human exposure to *Anopheles* bites in urban areas of Dakar region, Sénégal. Malaria Journal, 11, 72. 22424570 10.1186/1475-2875-11-72 PMC3337805

[12] Francischetti IM, Valenzuela JG, Pham VM, Garfield MK, Ribeiro JM. 2002. Toward a catalog for the transcripts and proteins (sialome) from the salivary gland of the malaria vector *Anopheles gambiae**.* Journal of Experimental Biology, 205(Pt 16), 2429–2451. 12124367 10.1242/jeb.205.16.2429

[13] Hewitt S, Delacollette C, Poirot E. 2013. Malaria control in the Greater Mekong Subregion: an overview of the current response and its limitations. Southeast Asian Journal of Tropical Medicine and Public Health, 44 (Suppl 1), 249–305; discussion 306–307. 24159835

[14] Hii J, Hustedt J, Bangs MJ. 2021. Residual malaria transmission in select countries of Asia-Pacific region: old wine in a new barrel. Journal of Infectious Diseases, 223(12 Suppl 2), S111–S142. 33906222 10.1093/infdis/jiab004 PMC8079134

[15] Jeyaprakasam NK, Liew JWK, Low VL, Wan-Sulaiman WY, Vythilingam I. 2020. *Plasmodium knowlesi* infecting humans in Southeast Asia: what’s next? PLoS Neglected Tropical Diseases, 14(12), e0008900. 33382697 10.1371/journal.pntd.0008900 PMC7774830

[16] Jongwutiwes S, Putaporntip C, Iwasaki T, Sata T, Kanbara H. 2004. Naturally acquired *Plasmodium knowlesi* malaria in human, Thailand. Emerging Infectious Diseases, 10(12), 2211–2213. 15663864 10.3201/eid1012.040293 PMC3323387

[17] Kassam NA, Kulaya N, Kaaya RD, Schmiegelow C, Wang CW, Kavishe RA, Alifrangis M.. 2021. Use of anti-gSG6–P1 IgG as a serological biomarker to assess temporal exposure to *Anopheles*’ mosquito bites in Lower Moshi. PLoS ONE, 16(10), e0259131. 34705869 10.1371/journal.pone.0259131 PMC8550589

[18] Lanfrancotti A, Lombardo F, Santolamazza F, Veneri M, Castrignanò T, Coluzzi M, Arcà B. 2002. Novel cDNAs encoding salivary proteins from the malaria vector *Anopheles gambiae**.* FEBS Letters, 517(1–3), 67–71. 12062411 10.1016/s0014-5793(02)02578-4

[19] Lê S, Josse J, Husson F. 2008. FactoMineR: an R package for multivariate analysis. Journal of Statistical Software, 25(1), 1–18.

[20] Lombardo F, Ronca R, Rizzo C, Mestres-Simòn M, Lanfrancotti A, Currà C, Fiorentino G, Bourgouin C, Ribeiro JM, Petrarca V, Ponzi M, Coluzzi M, Arcà B. 2009. The *Anopheles gambiae* salivary protein gSG6: An *Anopheline*-specific protein with a blood-feeding role. Insect Biochemistry and Molecular Biology, 39(7), 457–466. 19442731 10.1016/j.ibmb.2009.04.006 PMC3740408

[21] Londono-Renteria B, Drame PM, Weitzel T, Rosas R, Gripping C, Cardenas JC, Alvares M, Wesson DM, Poinsignon A, Remoue F, Colpitts TM. 2015. *An. gambiae* gSG6–P1 evaluation as a proxy for human-vector contact in the Americas: A pilot study. Parasites & Vectors, 8(1), 533. 26464073 10.1186/s13071-015-1160-3 PMC4605097

[22] Manguin S, Garros C, Dusfour I, Harbach RE, Coosemans M. 2008. Bionomics, taxonomy, and distribution of the major malaria vector taxa of *Anopheles* subgenus *Cellia* in Southeast Asia: an updated review. Infection, Genetics and Evolution, 8(4), 489–503. 10.1016/j.meegid.2007.11.004 18178531

[23] MoPH. 2025. Thailand Malaria Elimination Program. Nonthaburi: Division of Vector Borne Diseases. Ministry of Public Health. https://malaria.ddc.moph.go.th/malariar10/home.php.

[24] Morgan K, Somboon P, Walton C. 2013. Understanding *Anopheles* diversity in Southeast Asia and its applications for malaria control, in: *Anopheles* mosquitoes – new insights into malaria vectors, Manguin S, Ed. London, UK: IntechOpen. pp. 327–355.

[25] Ndo C, Elanga-Ndille E, Cheteug G, Metitsi RD, Wanji S, Moukoko CEE. 2022. IgG antibody responses to *Anopheles gambiae* gSG6–P1 salivary peptide are induced in human populations exposed to secondary malaria vectors in forest areas in Cameroon. PLoS One, 17(11), e0276991. 36355922 10.1371/journal.pone.0276991 PMC9648791

[26] Ngernna S, Rachaphaew N, Thammapalo S, Prikchoo P, Kaewnah O, Manopwisedjaroen K, Phumchuea K, Suansomjit C, Roobsoong W, Sattabongkot J, Cui L, Nguitragool W. 2019. Case report: Case series of human *Plasmodium knowlesi* infection on the southern border of Thailand. American Journal of Tropical Medicine and Hygiene, 101(6), 1397–1401. 31595871 10.4269/ajtmh.19-0063 PMC6896887

[27] Nguetsa GC, Elanga-Ndille E, Essangui Same EG, Nganso Keptchouang T, Mandeng SE, Ekoko Eyisap W, Binyang JA, Fogang B, Nouage L, Piameu M, Ayong L, Etang J, Wanji S, Eboumbou Moukoko CE. 2024. Utility of plasma anti-gSG6–P1 IgG levels in determining changes in *Anopheles gambiae* bite rates in a rural area of Cameroon. Scientific Reports, 14(1), 14294. 38906949 10.1038/s41598-024-58337-8 PMC11192751

[28] Poinsignon A, Cornelie S, Mestres-Simon M, Lanfrancotti A, Rossignol M, Boulanger D, Cisse B, Sokhna C, Arcà B, Simondon F, Remoue F. 2008. Novel peptide marker corresponding to salivary protein gSG6 potentially identifies exposure to *Anopheles* bites. PLoS One, 3(6), e2472. 18575604 10.1371/journal.pone.0002472 PMC2427200

[29] Pollard EJM, Patterson C, Russell TL, Apairamo A, Oscar J, Arcà B, Drakeley C, Burkot TR. 2019. Human exposure to *Anopheles farauti* bites in the Solomon Islands is not associated with IgG antibody response to the gSG6 salivary protein of *Anopheles gambiae*. Malaria Journal, 18(1), 334. 31570113 10.1186/s12936-019-2975-8 PMC6771112

[30] Pramasivan S, Ngui R, Jeyaprakasam NK, Liew JWK, Low VL, Mohamed Hassan N, Wan Sulaiman WY, Jaraee R, Abdul Rahman R, Jelip J, Vythilingam I. 2021. Spatial distribution of *Plasmodium knowlesi* cases and their vectors in Johor, Malaysia: in light of human malaria elimination. Malaria Journal, 20(1), 426. 34715864 10.1186/s12936-021-03963-0 PMC8555301

[31] Rattanarithikul R, Harrison BA, Harbach RE, Panthusiri P, Coleman RE, Panthusiri P. 2006. Illustrated keys to the mosquitoes of Thailand. IV. *Anopheles*. Southeast Asian Journal of Tropical Medicine and Public Health, 37(Suppl 2), 1–128. 17262930

[32] Rizzo C, Lombardo F, Ronca R, Mangano V, Sirima SB, Nèbiè I, Fiorentino G, Modiano D, Arcà B. 2014. Differential antibody response to the *Anopheles gambiae* gSG6 and cE5 salivary proteins in individuals naturally exposed to bites of malaria vectors. Parasites & Vectors, 7, 549. 25428638 10.1186/s13071-014-0549-8 PMC4253619

[33] Rizzo C, Ronca R, Fiorentino G, Verra F, Mangano V, Poinsignon A, Sirima SB, Nèbiè I, Lombardo F, Remoue F, Coluzzi M, Petrarca V, Modiano D, Arcà B. 2011. Humoral response to the *Anopheles gambiae* salivary protein gSG6: a serological indicator of exposure to Afrotropical malaria vectors. PLoS One, 6(3), e17980. 21437289 10.1371/journal.pone.0017980 PMC3060095

[34] Roh ME, Lausatianragit K, Chaitaveep N, Jongsakul K, Sudathip P, Raseebut C, Tabprasit S, Nonkaew P, Spring M, Arsanok M, Boonyarangka P, Sriwichai S, Sai-Ngam P, Chaisatit C, Pokpong P, Prempree P, Rossi S, Feldman M, Wojnarski M, Bennett A, Gosling R, Jearakul D, Lausatianragit W, Smith PL, Martin NJ, Lover AA, Fukuda MM. 2021. Civilian-military malaria outbreak response in Thailand: An example of multi-stakeholder engagement for malaria elimination. Malaria Journal, 20(1), 458. 34876133 10.1186/s12936-021-03995-6 PMC8650387

[35] Rosenberg R, Andre RG, Somchit L. 1990. Highly efficient dry season transmission of malaria in Thailand. Transactions of the Royal Society of Tropical Medicine and Hygiene, 84(1), 22–28. 2189240 10.1016/0035-9203(90)90367-n

[36] Saeung M, Aung PL, Jupatanakul N, Manguin S, Chareonviriyaphap T, Phuanukoonnon S. 2025. Estimating malaria risk behaviours and their determinants among at-risk populations in a pre-elimination setting, Sisaket Province, Thailand-Cambodia border. Malaria Journal, 24(1), 293. 41029671 10.1186/s12936-025-05558-5 PMC12487564

[37] Saeung M, Jupatanakul N, Afelt A, Suksirisawat K, Lhaosudto S, Ahebwa A, Hii J, Manguin S, Chareonviriyaphap T. 2025. Insights into spatio-temporal dynamics of *Anopheles* vectors while approaching malaria elimination along the Thailand-Cambodia border. Acta Tropica, 263, 107545. 39933646 10.1016/j.actatropica.2025.107545

[38] Saeung M, Jupatanakul N, Hii J, Thanispong K, Chareonviriyaphap T, Manguin S. 2025. Overview of national and local efforts to eliminate malaria in Thailand. Trends in Parasitology, 41(1), 52–65. 39694742 10.1016/j.pt.2024.11.006

[39] Saeung M, Pengon, Pethrak C, Thaiudomsup S, Lhaosudto S, Saeung A, Manguin S, Chareonviriyaphap T, Jupatanakul N. 2024. Dirus complex species identification PCR (DiCSIP) improves the identification of *Anopheles dirus* complex from the Greater Mekong Subregion. Parasites & Vectors, 17(1), 260. 38880909 10.1186/s13071-024-06321-6 PMC11181648

[40] Sagna AB, Sarr JB, Gaayeb L, Drame PM, Ndiath MO, Senghor S, Sow CS, Poinsignon A, Seck M, Hermann E, Schacht AM, Faye N, Sokhna C, Remoue F, Riveau G. 2013. gSG6–P1 salivary biomarker discriminates micro-geographical heterogeneity of human exposure to *Anopheles* bites in low and seasonal malaria areas. Parasites & Vectors, 6, 68. 23497646 10.1186/1756-3305-6-68 PMC3631127

[41] Saita S, Pan-Ngum W, Phuanukoonnon S, Sriwichai P, Silawan T, White LJ, Parker DM. 2019. Human population movement and behavioural patterns in malaria hotspots on the Thai-Myanmar border: implications for malaria elimination. Malaria Journal, 18(1), 64. 30849980 10.1186/s12936-019-2704-3 PMC6408830

[42] Sam J, Shamsusah NA, Ali AH, Hod R, Hassan MR, Agustar HK. 2022. Prevalence of simian malaria among macaques in Malaysia (2000–2021): A systematic review. PLoS Neglected Tropical Diseases, 16(7), e0010527. 35849568 10.1371/journal.pntd.0010527 PMC9292078

[43] Tainchum K, Kongmee M, Manguin S, Bangs MJ, Chareonviriyaphap T. 2015. *Anopheles* species diversity and distribution of the malaria vectors of Thailand. Trends in Parasitology, 31(3), 109–119. 25697632 10.1016/j.pt.2015.01.004

[44] Tananchai C, Manguin S, Bangs MJ, Chareonviriyaphap T. 2019. Malaria vectors and species complexes in Thailand: implications for vector control. Trends in Parasitology, 35(7), 544–558. 31182384 10.1016/j.pt.2019.04.013

[45] Vythilingam I. 2010. *Plasmodium knowlesi* in humans: a review on the role of its vectors in Malaysia. Tropical Biomedicine, 27(1), 1–12. 20562807

[46] Vythilingam I, Chua TH, Liew JWK, Manin BO, Ferguson HM.. 2021. The vectors of *Plasmodium knowlesi* and other simian malarias Southeast Asia: challenges in malaria elimination. Advances in Parasitology, 113, 131–189. 34620382 10.1016/bs.apar.2021.08.005

[47] Vythilingam I, Noorazian YM, Huat TC, Jiram AI, Yusri YM, Azahari AH, Norparina I, Noorrain A, Lokmanhakim S. 2008. *Plasmodium knowlesi* in humans, macaques and mosquitoes in peninsular Malaysia. Parasites & Vectors, 1(1), 26. 18710577 10.1186/1756-3305-1-26 PMC2531168

[48] WHO. 2014. Guidance note on the control of residual malaria parasite transmission. Geneva: World Health Organization

[49] WHO. 2015. Strategy for malaria elimination in the Greater Mekong Subregion (2015–2030). Geneva: World Health Organization.

[50] WHO. 2016. Eliminating malaria. Geneva: World Health Organization.

[51] WHO. 2017. A framework for malaria elimination. Geneva: World Health Organization.

[52] WHO. 2022. The Mekong malaria elimination programme accelerating malaria elimination in the Greater Mekong. Geneva: World Health Organization.

[53] WHO. 2023. World malaria report 2023. Geneva: World Health Organization.

[54] WHO. 2024. World malaria report 2024. Geneva: World Health Organization.

[55] WHO. 2025. World malaria report 2025. Geneva: World Health Organization.

[56] Wongkoon S, Jaroensutasinee M, Jaroensutasinee K. 2012. Assessing the temporal modelling for prediction of dengue infection in northern and north-eastern, Thailand. Tropical Biomedicine, 29(3), 339–348. 23018496

[57] Ya-Umphan P, Cerqueira D, Parker DM, Cottrell G, Poinsignon A, Remoue F, Brengues C, Chareonviriyaphap T, Nosten F, Corbel V. 2017. Use of an *Anopheles* salivary biomarker to assess malaria transmission risk along the Thailand-Myanmar border. Journal of Infectious Diseases, 215(3), 396–404. 27932615 10.1093/infdis/jiw543 PMC5853934

[58] Yanmanee S, Seethamchai S, Kuamsab N, Karaphan S, Suwonkerd W, Jongwutiwes S, Putaporntip C. 2023. Natural vectors of *Plasmodium knowlesi* and other primate, avian and ungulate malaria parasites in Narathiwat Province, Southern Thailand. Scientific Reports, 13(1), 8875. 37264067 10.1038/s41598-023-36017-3 PMC10235068

[59] Yongchaitrakul S, Singhasivanon P, Sudathip P, Sattabongkot J, Cui L, Prasittisuk C, Parker D, Phuanukoonnon S. 2022. Knowledge and practices regarding malaria and its prevention and their associated socio-demographic factors among those living along the Thailand-Myanmar border. Southeast Asian Journal of Tropical Medicine and Public Health, 53(6), 551–573.

