# Supplementary material for: Immunological surveillance using anti-gSG6-P1 IgG biomarker reveals spatio-temporal dynamics of Anopheles exposure and gaps in malaria risk assessment in the Greater Mekong Subregion
Source: Parasite. 2026 Mar 13;33:11. doi: 10.1051/parasite/2026012 (PMC12984028; doi:10.1051/parasite/2026012)
Supplement: Supplementary file 1 — Supplementary Methods: Detailed protocols for gSG6-P1 Antibody detection by enzyme-linked immunosorbent assay (ELISA) and FAMD. [file parasite-33-11-s1.pdf]

Supplementary information

Antibody detection by enzyme-linked immunosorbent assay (ELISA)

The 96-well ELISA plate (Life Sciences, Cat#3590) was coated with 0.2 µg of gSG6-P1 peptide (sequence: H-EKVWVDRDNVYCGHLDCTRVATF-OH, GENEPREP) in 100 µL 1X PBS per well (GENEPEP), while 100 µL of 0.1X blocking buffer (Thermo Fisher Scientific, Cat# 37570) in 1X PBS was used as a negative control. Each sample was conducted in technical duplicate. The plate was incubated at 37°C for 3 hrs in an incubator (Vision Scientific, VS-1203P3L) and washed three times with 200 µL/well of washing solution (0.1% Tween in distilled water). To prevent nonspecific antibody binding to the remaining surface, 200 µL of 0.1X blocking buffer diluted in 1X PBS was added to each well. The plate was incubated at 37°C for 3 hrs and washed three times with 200 µL/well of washing solution. Next,100 µL of eluted blood sample (diluted 1:10 in 1% Tween in 1X PBS) was added to each well, allowing the antibodies to bind to the gSG6-P1 peptide. The plate was incubated overnight at 4°C followed by three washes with 200 µL of washing solution per well. To detect bound antibodies, 100 µL of biotin-conjugated mouse anti-human IgG secondary antibody (0.05 µg/well, BD Biosciences, Cat#555785) diluted in 1% Tween in 1X PBS was added and incubated at 37°C for 1 hr and 30 mins followed by three washes with 200 µL/well of washing solution. Next, 100 µL of horseradish peroxidase (HRP)-conjugated streptavidin solution (0.002 µg/well, Jackson ImmunoResearch Laboratories, Cat#016-303-084) diluted in 1% tween in 1X PBS & was added to each well and incubated at 37°C for 1 hour, followed by a final washing step.

For signal detection, 100 µL of substrate solution containing 0.1 mg 2,2'-azino-bis (3-ethylbenzothiazoline-6-sulfonic acid) (ABTS, Sigma-Aldrich, Cat# A9941-100TAB) and 40% H_2O_2 in 0.05M citrate buffer, pH4 was added to each well. The plate was wrapped in aluminum foil and incubated at room temperature for 1 hr. The optical density (OD) was measured at 410 nm using an ELISA plate reader (BioTek, Synergy H1).

The absolute antibody levels obtained from each well were corrected by subtracting the noise value (negative control). The corrected values were expressed as delta optical density (ΔOD) and used to calculate the average antibody level for each sample. To account for plate-to-plate variability, ΔOD values were normalized using standard curves generated from five blood samples representing a range of antibody responses. This normalization approach enhanced the reliability and comparability of results across multiple assay plates.

Factor Analysis for Mixed Data (FAMD)

FAMD is a variant of Multiple Factor Analysis that integrates principal component analysis for continuous variables and multiple correspondence analysis for categorical variables, allowing for the simultaneous analysis of both data types [1]. For this analysis, three categorical variables (season, village, and distance from house to rubber plot) and two continuous variables (frequency of entering rubber plot per week (RubberPerWeek), and age of volunteers) were included. Prior to analysis, antibody levels were ranked in ascending order and classified into five groups based on the number of data points, with the average antibody level calculated for each group. FAMD was used to visualize variables in a multi-dimensional space, capturing their interactions. The first two dimensions, which explained the majority of data variance, were selected for further analysis. Variables within each dimension were assessed based on their contribution values, with those exceeding the expected average considered significant contributors to the principal dimensions. A FAMD factor map was then plotted to observe patterns in antibody levels and identify key influencing variables.

Reference

1. Pagès, J. Analyse factorielle de données mixtes. Rev Stat Appliquée. 2004; 52(4): p. 93-111.
