# Supplementary material for: Immunological surveillance using anti-gSG6-P1 IgG biomarker reveals spatio-temporal dynamics of Anopheles exposure and gaps in malaria risk assessment in the Greater Mekong Subregion
Source: Parasite. 2026 Mar 13;33:11. doi: 10.1051/parasite/2026012 (PMC12984028; doi:10.1051/parasite/2026012)
Supplement: Supplementary file 2 — Supplementary Table 1. Characteristics of the 184 participants from Sisaket Province (age 18–60 years old), from August 2022 to April 2023. [file parasite-33-11-s2.pdf]

Supplementary Table 1. Characteristics of the 184 participants from Sisaket Province (age 18–60 years old), from August 2022 to April 2023

**Characteristics**	**Huai Chan**		**Kan Throm Tai**		**Non Thon Lang**	
	**n = 59**	**32.07%**	**n = 97**	**52.72%**	**n = 28**	**15.22%**
**Gender**						
Male	24	13.04%	35	19.02%	9	4.89%
Female	35	19.02%	62	33.70%	19	10.33%
**Age**						
18–30 years	12	6.52%	9	4.89%	2	1.09%
31–40 years	11	5.98%	23	12.50%	3	1.63%
41–50 years	21	11.41%	34	18.48%	8	4.35%
51–60 years	15	8.15%	31	16.85%	15	8.15%
**Distance between house and rubber plot**						
<1 KM	2	1.09%	0	0%	4	2.17%
1–5 KM	27	14.67%	30	16.30%	19	10.33%
>5 KM	30	16.30%	67	36.41%	5	2.72%
**RubberPerWeek**						
**Rainy**						
0–4 day/week	21	11.41%	88	47.83%	20	10.87%
5–7 day/week	38	20.65%	9	4.89%	8	4.35%
**Cool-dry**						
0–4 day/week	31	16.85%	40	21.74%	9	4.89%
5–7 day/week	28	15.22%	57	30.98%	19	10.33%
**Hot-dry**						
0–4 day/week	44	23.91%	25	13.59%	16	8.70%
5–7 day/week	15	8.15%	72	39.13%	12	6.52%
